# The Corrected Serum Sodium Concentration in Hyperglycemic Crises: Computation and Clinical Applications

**DOI:** 10.3389/fmed.2020.00477

**Published:** 2020-08-25

**Authors:** Todd S. Ing, Kavitha Ganta, Gautam Bhave, Susie Q. Lew, Emmanuel I. Agaba, Christos Argyropoulos, Antonios H. Tzamaloukas

**Affiliations:** ^1^Department of Medicine, Stritch School of Medicine, Loyola University Chicago, Chicago, IL, United States; ^2^Medicine Service, Department of Medicine, Raymond G. Murphy Veterans Affairs Medical Center, University of New Mexico School of Medicine, Albuquerque, NM, United States; ^3^Department of Medicine, Vanderbilt University Medical Center, Nashville, TN, United States; ^4^Department of Medicine, George Washington University School of Medicine, Washington, DC, United States; ^5^Department of Medicine, University of Jos, Jos, Nigeria; ^6^Department of Medicine, University of New Mexico School of Medicine, Albuquerque, NM, United States; ^7^Research Service, Department of Medicine, Raymond G. Murphy Veterans Affairs Medical Center, University of New Mexico School of Medicine, Albuquerque, NM, United States

**Keywords:** sodium concentration, hyperglycemia, dysnatremia, hypertonicity, diabetic ketoacidosis, hyperosmolar hyperglycemia

## Abstract

In hyperglycemia, hypertonicity results from solute (glucose) gain and loss of water in excess of sodium plus potassium through osmotic diuresis. Patients with stage 5 chronic kidney disease (CKD) and hyperglycemia have minimal or no osmotic diuresis; patients with preserved renal function and diabetic ketoacidosis (DKA) or hyperosmolar hyperglycemic state (HHS) have often large osmotic diuresis. Hypertonicity from glucose gain is reversed with normalization of serum glucose ([Glu]); hypertonicity due to osmotic diuresis requires infusion of hypotonic solutions. Prediction of the serum sodium after [Glu] normalization (the corrected [Na]) estimates the part of hypertonicity caused by osmotic diuresis. Theoretical methods calculating the corrected [Na] and clinical reports allowing its calculation were reviewed. Corrected [Na] was computed separately in reports of DKA, HHS and hyperglycemia in CKD stage 5. The theoretical prediction of [Na] increase by 1.6 mmol/L per 5.6 mmol/L decrease in [Glu] in most clinical settings, except in extreme hyperglycemia or profound hypervolemia, was supported by studies of hyperglycemia in CKD stage 5 treated only with insulin. Mean corrected [Na] was 139.0 mmol/L in 772 hyperglycemic episodes in CKD stage 5 patients. In patients with preserved renal function, mean corrected [Na] was within the eunatremic range (141.1 mmol/L) in 7,812 DKA cases, and in the range of severe hypernatremia (160.8 mmol/L) in 755 cases of HHS. However, in DKA corrected [Na] was in the hypernatremic range in several reports and rose during treatment with adverse neurological consequences in other reports. The corrected [Na], computed as [Na] increase by 1.6 mmol/L per 5.6 mmol/L decrease in [Glu], provides a reasonable estimate of the degree of hypertonicity due to losses of hypotonic fluids through osmotic diuresis at presentation of DKH or HHS and should guide the tonicity of replacement solutions. However, the corrected [Na] may change during treatment because of ongoing fluid losses and should be monitored during treatment.

## Introduction

Imbalances that develop in patients with severe hyperglycemia and preserved renal function include extracellular gain of solute (glucose) and deficits of water, sodium, potassium, and other ions resulting from glycosuria. These imbalances, which cause extracellular and intracellular volume deficits, changes in the concentrations of key serum ions, and hypertonicity, constitute major treatment targets (1).

This report addresses the management of hyperglycemic hypertonicity. Hypertonicity is seen routinely in severe hyperglycemia (1–5), causes potentially life-threatening neurological manifestations (6, 7), and represents an important component of the treatment (1, 2).

## Corrected Sodium Level, Glucose Concentration, and Tonicity

In experimental studies, tonicity of a fluid can be measured directly by rapid photographic recordings of changes in the volume of cells, usually red blood cells, suspended in the fluid of interest (8). In clinical practice, tonicity is evaluated by surrogate biochemical measurements, including serum osmolality and sodium concentration ([Na]) (8). In the absence of solutes with extracellular distribution other than sodium salts (e.g., glucose), [Na] represents the index expressing tonicity (8). In hyperglycemia, glucose accumulation in the extracellular compartment contributes to tonicity (Ton), which is expressed by the formula (9):

(1) $$\begin{array}{l} Ton=2\times \left[Na\right]+\left[Glu\right]\;mOsm/L \end{array}$$

where the serum glucose concentration ([Glu]) is in mmol/L (1 mmol/L = 18 mg/dL).

Formula 1 provides accurate information on tonicity in hyperglycemia, except when high levels of plasma solids lower plasma water content (e.g., in hyperlipidemia) causing falsely low measurement of [Na] by indirect potentiometry (10). However, this formula should not be used to guide the composition of the replacement solution. Hypertonicity in hyperglycemia results from gain of extracellular solute (glucose) and osmotic diuresis (2, 5). Correction of hyperglycemia results in extracellular solute loss (11) and decrease in tonicity (12). The tonicity of the replacement solutions should correct the component of hypertonicity resulting from osmotic diuresis (2, 5). [Na] rises during correction of hyperglycemia as water is transferred osmotically from the extracellular into the intracellular compartment (12). The tonicity of replacement solutions should be based on the projected value of [Na] after normalization of [Glu] (2, 6, 8). The corrected [Na] is calculated using a predicted value of the change in [Na] (Δ[Na]) that results directly from the required change in [Glu] (Δ[Glu]) and is applied in the evaluation of the component of hyperglycemic hypertonicity that results from osmotic diuresis. The value of the coefficient used to calculate the corrected [Na] is a point of dispute (13).

This perspective article reviews the sources of various estimates of the coefficient for the corrected [Na] and clinical studies providing evidence for the appropriate coefficient. Then, it computes the corrected [Na] in reports of various categories of hyperglycemic crises, and based on these last reports, provides a frame for the clinical application of the corrected [Na].

## Derivations of the Corrected [Na] in Hyperglycemia

### Closed System of Hyperglycemia: Theoretical Calculations

This section addresses the modeling of the effect on [Na] from change in [Glu] not accompanied by changes in external balance of body water or monovalent cations, i.e., in a closed system. The theoretical calculations explore the principle asserting that in hyperglycemia [Glu] represents the ratio (baseline amount of extracellular glucose plus amount of glucose gained)/ECFV, where ECFV is hyperglycemic extracellular volume, while Ton (formula 1) represents the ratio (baseline effective body solute plus amount of glucose gained)/TBW, where TBW is total body water (7). Applying this principle and considering that the amounts of sodium in the extracellular compartment and effective solute in the intracellular compartment remain constant during development of hyperglycemia, Katz calculated that [Na] changes by 1.6 mmol/L in the opposite direction of every 5.6 mmol/L (100 mg/dL) change in [Glu] (Δ[Na]/Δ[Glu]) = −1.6 mmol/L per 5.6 mmol/L) (14).

Goldberg proposed using the Katz coefficient to predict the value of [Na] after correction of hyperglycemia (15). Subsequently, Al-Kudsi et al. provided the following formula to calculate this corrected [Na] (16):

(2) $$\begin{array}{l} Corrected\;\left[Na\right]=\;Actual\;\left[Na\right]\\ \;+1.6\times \frac{\left[Glu\right]-\;5.6\frac{mmol}{L}\;\left(or\;100\frac{mg}{dL}\;\right)}{5.6\;\left(or\;100\right)} \end{array}$$

The Al-Kudsi formula predicts the value of [Na] after correction of [Glu] to 5.6 mmol/L (100 mg/dL) without any change in body water, sodium or potassium (17). The corrected [Na] at any desired final value of [Glu] can be calculated by substituting this desired [Glu] for 5.6 mmol/L in the numerator of the Al-Kudsi formula.

Δ[Na]/Δ[Glu] values numerically lower than 2.8 mmol/L per 5.6 mmol/L indicate rise in tonicity, while values numerically higher than 2.8 indicate decrease in tonicity during development of hyperglycemia. The Katz report created new insights into the change in tonicity produced by glucose gain. Prior to this report, the value of Δ[Na]/Δ[Glu] used by clinicians, at −2.8 mmol/L per 5.6 mmol/L (18), indicated no change in tonicity as [Glu] rises because a rise in [Glu] by 5.6 mmol/L would be completely offset by a 2.8 mmol/L decrease in [Na] (formula 1). This Δ[Na]/Δ[Glu] value stemmed from the faulty premise that osmotic transfer of intracellular water into the extracellular space as a result of extracellular glucose gain was not associated with an increase in intracellular tonicity. As a matter of fact, the increase in total body effective solute (baseline solute plus glucose gain) causes equal rises in both intracellular and extracellular fluid tonicities. The glucose-induced gain in extracellular solute causes water exit from the cells (19) to bring about hypertonic hyponatremia (20). Katz's coefficient computes tonicity increase (ΔTon) of 2.4 (5.6 – 2 ×1.6) mOsm/L for every 5.6 mmol/L increase in [Glu] (2).

Several guidelines for managing hyperglycemia (21–24) and other reports (25–29) have adopted the Katz coefficient for calculating the corrected [Na]. Alternate guidelines for treating hyperglycemia (30, 31) and hyponatremia (32), and various other reports (33–35) advocate other coefficients. The proposed correction coefficients for [Na] range between −1.35 and −4.0 mmol/L per 5.6 mmol/L Δ[Glu] (36). The variation of these coefficients resulted from both theoretical calculations and clinical studies. This section addresses the theoretical calculations. All theoretical calculations of Δ[Na]/Δ[Glu] are based on the principle stating that in hyperglycemia the volume determining Δ[Glu] is ECFV_2_ while the volume determining the rise in tonicity is TBW (7).

Calculations based on the same principles as those applied by Katz revealed that Δ[Na]/Δ[Glu] can obtain several values in a closed system (37–40). In these calculations, the important influences on Δ[Na], Δ[Glu], Δ[Na]/Δ[Glu] derive from the degree of hyperglycemia (38) and the state of euglycemic extracellular volume (ECFV_1_) (39), or more specifically the glucose gained per liter of ECFV_1_ ([Glu]_A_) (17, 40) and the baseline, at euglycemia, ratio of intracellular volume (ICFV_1_) to ECFV_1_ (the volume ratio α_1_) (17, 39, 40). Note: In the text and Table 1, the subscripts 1 and 2 denote respectively baseline euglycemia and hyperglycemia.

**Table 1:** Tonicity-related and body fluid variables in a closed system of hyperglycemia.

Total body water_1_	*ICFV*_1_ + *ECFV*_1_
Total extracellular glucose_1_	*ECFV*_1_ × [*Glu*]_1_
Total extracellular sodium_1_	*ECFV*_1_ × [*Na*]_1_
${\text{Total extracellular solute}}_{1}^{*}$	*ECFV*_1_ × ([*Glu*]_1_ + 2 × [*Na*]_1_)
${\text{Total intracellular solute}}_{1}^{*}$	α_1_ × *ECFV*_1_ × ([*Glu*]_1_ + 2 × [*Na*]_1_)
Total glucose gain_2_	*ECFV*_1_ × [*Glu*]_*A*_
Total extracellular glucose_2_	*ECFV*_1_ × ([*Glu*]_1_ + [*Glu*]_*A*_)
${\text{Total extracellular solute}}_{2}^{*}$	*ECFV*_1_ × ([*Glu*]_1_ + [*Glu*]_*A*_ + 2 × [*Na*]_1_)
Volume ratio α_2_	$\frac{Total\;intracellular\;solute_{1}*}{Total\;extracellular\;solute_{2}*}$
ECFV_2_	(*ECFV*_1_ + *ICFV*_1_)/(α_2_ + 1)
[Glu]_2_	*ECFV*_1_ × ([*Glu*]_1_ + [*Glu*]_*A*_)/*ECFV*_2_
[Na]_2_	*ECFV*_1_ × [*Na*]_1_/*ECFV*_2_
Δ[Na]/Δ[Glu]	{([*Na*]_2_ − [*Na*]_1_)/([*Glu*]_2_ − [*Glu*]_1_)} ×5.6
${\text{Ton}}_{2}^{*}$	${\left[Glu\right]}_{2}+2\times {\left[Na\right]}_{2},\;or\frac{Total\;intracellular\;solute_{1}+Total\;extracellular\;solute_{2}}{ICFV_{1}+ECFV_{1}}$
ΔTon	*Ton*_2_−*Ton*_1_, or $\frac{ECFV_{1\;}x{\left[Glu\right]}_{A}}{ICFV_{1}+ECFV_{1}},\;$ or [*Glu*] + 2 × [*Na*]^**^

*ICFV, intracellular fluid volume; ECFV, extracellular fluid volume, [Na], serum sodium concentration; [Glu], serum glucose concentration in mmol/L; Ton, tonicity; subscript 1, baseline euglycemic state; subscript 2, hyperglycemic state; [Glu]_A_, glucose gained per liter of euglycemic ECFV*.

* *Solutes distributed in total body water (e.g., urea) are not included in these formulas because changes in their body content do not cause osmotic fluid shifts between the intracellular and extracellular compartments (i.e., changes in tonicity)*.

** *2 × Δ[Na] has a negative value in this equation, since Δ[Na] = [Na]_2_ – [Na]_1_ and [Na]_2_ < [Na]_1_*.

The ICVF/ECVF volume ratio α increases during development of hypovolemia or correction of hyperglycemia and decreases during development of hypervolemia or of hyperglycemia. Total glucose gain during development of hyperglycemia is the product of ECFV_1_ and [Glu]_A_. [Glu]_A_, which is not a glucose concentration encountered clinically because ECFV increases during development of hyperglycemia, was entered in the calculations because the same [Glu]_A_ leads to comparable [Glu]_2_ values in hypothetical subjects with widely different euglycemic volume ratio α_1_ (17).

Table 1 shows general formulas used by Katz for computation of tonicity-related and volume-related parameters. Total intracellular and extracellular solutes in this Table are the total solutes determining tonicity (14). Solutes with body water distribution, e.g., urea, do not contribute to tonicity (8, 9) and are not included in the calculations of the tonicity-related variables (9, 14). The formula for calculating the hyperglycemic ICFV_2_/ECFV_2_ ratio α_2_ in Table 1 as the corresponding ratio of effective intracellular-to-extracellular solute is based on the principle asserting that body water is apportioned between the intracellular and extracellular compartments in proportion to the amount of solute in each compartment (41). The calculations for the various Δ[Na]/Δ[Glu] coefficients in hyperglycemia (37–40) and for the following examples were based on the formulas of Table 1. Theoretical examples will show the effects of the ICFV_1_/ECFV_1_ volume ratio α_1_ and of the degree of hyperglycemia on tonicity-related parameters in hypothetical patients. For all examples the baseline values were 5.6 mmol/L for [Glu]_1_, 140 mmol/L per [Na]_1_, and 285.6 mOsm/L for Ton_1_.

For the same volume ratio α_1_, the same degree of hyperglycemia results in the same hypertonicity values regardless of the size of extracellular volume. Example: Two hypothetical patients, with TBW 48 L and 92 L and both with α_1_ = 2.0, as in Katz's report (14), and [Glu]_A_ = 100 mmol/L. ECFV_1_ values are 16 and 32 L and glucose loads are 1,600 (16 ×100) and 3,200 (32 ×100) mmol, respectively. In both patients, [Glu]_2_ = 87.3 mmol/L (1,571 mg/dL), [Na]_2_ = 115.7 mmol/L, Δ[Na]/Δ[Glu] = −1.66 mmol/L per 5.6 mmol/L, and Ton_2_ = 318.7 mOsm/L.

For comparable degrees of hyperglycemia, hypertonicity is higher in hypervolemia and lower in hypovolemia compared to euvolemia. [Glu]_A_ was 100 mmol/L in the euvolemic, hypervolemic, and hypovolemic examples. Euvolemic values are shown in the previous example. A hypothetical patient with TBW = 64 L, α_1_ = 1.0, ECFV_1_ = 32 L, and glucose load 3,200 (32 ×100) mmol represents severe hypervolemia. In this patient, [Glu]_2_ = 91.8 mmol/L (1,653 mg/dL), [Na]_2_ = 127.9 mmol/L, Δ[Na]/Δ[Glu] = −1.18 mmol/L per 5.6 mmol/L, and Ton_2_ = 335.6 mOsm/L. Another hypothetical patient with TBW = 40 L, α_1_ = 4.0, ECFV_1_ = 8 L, and glucose load 800 (8 ×100) mmol represents severe hypovolemia. In this patient, [Glu]_2_ = 83.8 mmol/L (1,509 mg/dL), [Na]_2_ = 111.1 mmol/L, Δ[Na]/Δ[Glu] = −2.10 mmol/L per 5.6 mmol/L, and Ton_2_ = 305.6 mOsm/L.

Although the rises in tonicity are progressively equal at progressive equal glucose loads, the increases in the [Glu] and the decreases in both [Na] and Δ[Na]/Δ[Glu] decrease progressively (38). The change in Δ[Na]/Δ[Glu] at progressive hyperglycemia is very gradual. For example, in the hypothetical patient with α_1_ = 2.0, the rate of rise in tonicity for each 100 mmol/L of [Glu]_A_ is 33.3 mOsm/L; for the first [Glu]_A_ of 100 mmol/L, [Glu] rises by 81.7 mmol/L from 5.6 to 87.3 mmol/L and [Na] decreases by 24.2 mmol/L from 140 to 115.8 mmol/L for a Δ[Na]/Δ[Glu] of −1.66 mmol/L per 5.6 mmol/L; for a subsequent [Glu]_A_ of another 100 mmol/L, [Glu] rises by 60.2 mmol/L from 87.3 to 149.1 mmol/L (2,684 mg/dL), and [Na] decreases by 14.2 mmol/L from 115.8 to 101.6 mmol/L for a Δ[Na]/Δ[Glu] of −1.29 mmol/L per 5.6 mmol/L in this step and −1.50 mmol/L per 5.6 mmol/L from the euglycemic state.

In addition to the degree of hyperglycemia and the volume ratio α_1_, the following potential sources of variations in Δ[Na]/Δ[Glu] and therefore in corrected [Na] have been discussed: (a) differences in the apparent volumes of distribution of glucose and sodium due to intracellular entry of sodium ions, which is larger in some chronic illnesses than in the normal state (17), or to absence of insulin requirement for glucose uptake by the cells of certain organs, which was the source of the proposed value of −1.35 mmol/L per 5.6 mmol/L for Δ[Na]/Δ[Glu] (37). The difference in corrected [Na]_2_ values calculated with Δ[Na]/Δ[Glu] values of −1.6 and −1.35 mmol/L per 5.6 mmol/L has little clinical significance even in extreme hyperglycemia. For example, if [Na]_1_ is 140 mmol/L at a [Glu]_1_ of 5.6 mmol/L (100 mg/dL), [Na]_2_ values calculated, respectively by Δ[Na]/Δ[Glu] of −1.6 and −1.35 mmol/L per 5.6 mmol/L are 109.6 and 114.4 mmol/L if [Glu] rises to 111.1 mmol/L (2,000 mg/dL). (b) Differences in sodium concentration between plasma and interstitial compartment due to Gibbs-Donnan equilibrium between these two sub-compartments of the ECFV (17); (c) Exit of potassium from cells and changes in intracellular solute during development of hyperglycemia (39). Potassium transfer may alter the value of Δ[Na]/Δ[Glu], but this effect will be small in hyperglycemia developing in a closed system (17). (d) Sodium exchanges between sodium stores in proteoglycans (mainly glycosaminoglycan) in skin, bones and cartilage (42) and sodium in the extracellular compartment, which could influence Δ[Na]/Δ[Glu], but have not been studied in hyperglycemia (17).

Finally, both definition and methods of measurement of ECFV encounter difficulties (43, 44). Nevertheless, the average normal ECFV is in the order of 40–45% of TBW, not 33% as in Katz's calculations (43, 44), and therefore at euvolemia the ICFV_1_/ECFV_1_ volume ratio α_1_ should be between 1.50 and 1.22, not 2.00. For a [Glu]_A_ of 100 mmol/L, Δ[Na]/Δ[Glu] is −1.44 mmol/L per 5.6 mmol/L if α_1_ is 1.22. In a patient presenting with [Glu]_2_ = 105.6 mmol/L (1,100 mg/dL) and [Na]_2_ = 111.4 mmol/L, the corrected [Na], for Δ[Glu] = 100 mmol/L, is 140.0 mmol/L using the Katz coefficient for Δ[Na]/Δ[Glu] of −1.6 and 137.1 mmol/L using a Δ[Na]/Δ[Glu] of −1.22 mmol/L per 5.6 mmol/L. The difference between these corrected [Na] values has minimal clinical significance.

### Closed System of Hyperglycemia: Clinical Observations

Hyperglycemia in patients with advanced renal failure allows study of the theoretical predictions in a closed system because it can be treated with insulin infusion and with no or minimal changes in the external balance of sodium, potassium and water (45, 46). One report analyzed 43 episodes of severe hyperglycemia ([Glu] > 33.3 mmol/L or 600 mg/dL), treated with insulin and no other interventions, in patients on chronic dialysis with no or minimal fluid intake and urine loss and no change in body weight during treatment (47). Mean ± standard deviation values at presentation and end of observation, respectively, were as follows: [Glu] 50.7 ± 10.9 mmol/L (913 ± 197 mg/dL) and 9.4 ± 4.3 mmol/L (170 ± 78 mg/dL); [Na] 125 ± 5 and 136 ± 5 mmol/L; and Ton 300 ± 13 and 282 ± 11 mOsm/L. Δ[Na]/Δ[Glu] was −1.50 ± 0.22 mmol/L per 5.6 mmol/L. In a review, which analyzed 148 hyperglycemic episodes in patients on dialysis, Δ[Na]/Δ[Glu] was −1.61 ± 0.36 mmol/L per 5.6 mmol/L (48). In both reports, a small number of patients with pronounced edema had Δ[Na]/Δ[Glu] values numerically much lower than 1.6 per 5.6 mmol/L in agreement with theoretical predictions (47, 48); no episode of extreme hyperglycemia with Δ[Na]/Δ[Glu] substantially different from 1.6 mmol/L per 5.6 mmol/L was observed.

Another study analyzed the relationship between [Glu] and [Na] by linear regression in 208 patients on dialysis who had at least three measurements of [Glu] and [Na] and a difference between the lowest and highest value of [Glu] exceeding 16.7 mmol/L (300 mg/dL). In this study, the 5th−95th percentile range of [Glu] was 4.5–30.5 mmol/L (81–549 mg/dL) and Δ[Na]/Δ[Glu] was −1.47±0.82 mmol/L per 5.6 mmol/L (49).

The reports of hyperglycemic episodes in dialysis patients provided support for Katz's Δ[Na]/Δ[Glu] coefficient of −1.6 mmol/L per 5.6 mmol/L for computation of the corrected [Na] in oligoanuric patients. Hyperglycemic episodes in patients with preserved renal function represent a different entity. The next section addresses these patients.

### Open System of Hyperglycemia: Theoretical Calculations

Severe hyperglycemia in patients with preserved renal function causes deficits in body sodium, potassium, and water, which are the key determinants of [Na] at euglycemia (50). Balance abnormalities specific to hyperglycemia develop from water gain in the gastrointestinal tract and losses of water, sodium and potassium from the urinary tract. Thirst is caused by hyperglycemic hypertonicity and hypovolemia from urinary losses. Hyperglycemic hypertonicity caused thirst in animal experiments (51). Polydipsia is a prominent clinical manifestation of hyperglycemic crises (7, 52, 53). Water intake from hyperglycemia led to hyponatremia after correction with insulin of approximately one-third of the hyperglycemic episodes in dialysis patients (47).

A major rise in tonicity in hyperglycemia results from osmotic diuresis, in which water loss is relatively greater than loss of sodium plus potassium (5, 17, 54). Thus, in hyperglycemic crises occurring in patients with preserved renal function, who represent an open system, [Na] receives influences from three pathophysiologic processes: rise in [Glu] and water gain cause [Na] decreases, while osmotic diuresis causes [Na] increase. In these patients, quantitating the isolated effect of glucose gain is imperative because this effect is predictable with a reasonable degree of certainty, as shown in the previous section, and more importantly, it will disappear with correction of hyperglycemia without requiring additional measures.

Prediction of the quantitative effects of water intake and particularly of osmotic diuresis, which is the dominant effect on tonicity in severe hyperglycemic episodes (2, 3), is difficult because the magnitude of these processes varies greatly (2, 17). The effects of osmotic diuresis on [Na] require correction by fluid infusion. One report calculated the effects of osmotic diuresis on tonicity-related values in a hypothetical subject with extreme hyperglycemia ([Glu] of 137.9 mmol/L or 2,483 mg/dL) by using the average reported values of urinary water loss (25% of baseline body water) and urinary sodium plus potassium concentration (60 mmol/L) in severe hyperglycemia (17). In this hypothetical subject, [Na] after correction of hyperglycemia calculated from the body contents of water, glucose, sodium, and potassium at baseline and after their changes from osmotic diuresis, was 167 mmol/L, while corrected [Na] calculated by the Al-Kudsi formula was 169 mmol/L. This finding suggests that the corrected [Na] by the Al-Kudsi formula provides a reasonable prediction of the part of hypertonicity that is due to osmotic diuresis.

### Open System of Hyperglycemia: Clinical Observations

Accounting for changes in external balances of water, sodium, and potassium during development and treatment of hyperglycemia is necessary for any evaluation of the corrected [Na] in patients with renal function. There is a paucity of studies in this area. In a prospective study, hyperglycemia was produced in normal volunteers by infusion of somatostatin and 20% dextrose in 0.45% saline and the relation between [Glu] and [Na] was evaluated by linear regression (55); the overall slope of Δ[Na]/Δ[Glu was −2.4 mmol/L per 5.6 mmol/L. However, in a piecewise linear regression, this slope was −1.6 mmol/L per 5.6 mmol/L up to a [Glu] of 24.4 mmol/L (440 mg/dL) and −4.0 mmol/L per 5.6 mmol/L between [Glu] levels of 24.4 and 44.4 mmol/L (440–800 mg/dL). These findings were used in the development of several guidelines (30–32).

Assuming baseline values of 5.6 mmol/L for [Glu], 140 mmol/L for [Na], and 285.6 mOsm/L for tonicity, we calculated Ton_2_ values in hyperglycemia using the Δ[Na]/Δ[Glu] slopes −1.6 and −4.0 per 5.6 mmol/L (53). A slope of −1.6 mmol/L per 5.6 mmol/L for [Glu] between 5.6 and 24.4 mmol/L, calculates at a [Glu]_2_ of 24.4 mmol/L a [Na]_2_ of 134.6 mmol/L and a Ton_2_ 293.5 mOsm/L (formula 1). A slope of −4.0 mmol/L per 5.6 mmol/L between [Glu] 24.4 and 44.4 mmol/L, calculates at a [Glu]_2_ of 44.4 mmol/L a [Na]_2_ of 120.2 mmol/L and a Ton_2_ of 284.8 mOsm/L. According to these calculations, tonicity, after rising appropriately with [Glu] rising from 5.6 to 24.4 mmol/L, decreased to its baseline euglycemic level after further [Glu] rise to 44.4 mmol/L. As noted earlier, Δ[Na]/Δ[Glu] values numerically larger than 2.8 mmol/L per 5.6 mmol/L are inconsistent with the principle of rise in tonicity as [Glu] rises. Consequently, the Δ[Na]/Δ[Glu] value of −4.0 mmol/L per 5.6 mmol/L could not be the exclusive result of a rise in [Glu] from 24.4 to 44.4 mmol/L. Water gain added to development of hyperglycemia is the most probable mechanism for this Δ[Na]/Δ[Glu] value. Another prospective study of rising [Glu] in patients with diabetes mellitus computed a Δ[Na]/Δ[Glu of −1.50 mmol/L per 5.6 mmol/L (56).

### Clinical Application of the Corrected [Na]: Definition of the Hyperglycemic Syndromes

The guidelines for hyperglycemic crises address diabetic ketoacidosis (DKA) and hyperosmolar hyperglycemic state (HHS) (1, 21–24). The diagnostic features of DKA include low arterial blood pH and serum bicarbonate, presence of ketone bodies in serum and urine, a wide serum anion gap, and variable tonicity (1). [Glu] > 13.9 mmol/L (250 mg/dL) or > 11.1 mmol/L (200 mg/dL) was also included in these criteria (1, 21). However, euglycemic DKA has become more frequent after the introduction of sodium glucose cotransporter 2 (SGLT-2) inhibitors in the treatment of diabetes mellitus (57).

The criteria for diagnosis of HHS include Ton ≥ 320 mOsm/L, [Glu] ≥ 33.3 mmol/L (600 mg/dL) and serum bicarbonate ≥ 15 mmol/L (1, 20, 58), or Ton ≥ 330 mOsm/L (59) and [Glu] ≥ 30 mmol/L (540 mg/dL) (27, 59). Hypertonicity may cause coma in hyperglycemic syndromes (60, 61). Ton ≥ 320 mOsm/L causes coma frequently (52), although neurological manifestations from hypertonicity may occur in hyperglycemic patients with lower tonicity levels (62, 63).

At equal levels of hyperglycemic hypertonicity, elevated [Na] indicates severe water deficit (64, 65). The corrected [Na] illustrates the difference in water deficit between high [Na] and high [Glu] in this case. For example, for a given Ton of 320 mOsm/L, if [Glu] is 30 mmol/L (540 mg/dL) [Na] will be 145 mmol/L and the corrected [Na], at 152 mmol/L, indicates a water deficit, relative to the effective solute state, of 8.5% (= {152/140} × 100), while if [Glu] is 90 mmol/L (1,620 mg/dL), [Na] will be 115 mmol/L and the corrected [Na], at 141 mmol/L, indicates essentially similar status of body water and body effective solute.

### Clinical Application of the Corrected [Na]: Corrected [Na] in Hyperglycemic Syndromes

Table 2 shows presenting values for [Glu], [Na], tonicity, and corrected [Na] in reports of DKA (66–171), HHS (3, 9, 13, 75–78, 110, 129, 169, 172–251), and hyperglycemia in chronic kidney disease (CKD) stage V (12, 16, 47–49, 156, 171, 252–277), which was included in Table 2 as the control group because it causes limited or no water and electrolyte losses through osmotic diuresis. All but three of the cases in this last group were on maintenance dialysis. To show the range of the tonicity-related values, Table 2 includes studies as well as case reports. Reports of combined DKA and HHS were included in the DKA part of the table. Studies reporting median, instead of mean, tonicity-related values were not included in this table. The DKA category does not include reports on euglycemic DKA, while the HHS category contains six cases with Ton ≥ 320 mOsm/L and [Glu] between 17.2 mmol/L (310 mg/dL) and 30 mmol/L (540 mg/dL). The reason for including these cases was explained above. The corrected [Na] was calculated de novo in reports that did not calculate the corrected [Na] and was recalculated using the Al-Kudsi formula in reports that had used Δ[Na]/Δ[Glu] coefficients different from −1.6 mmol/L per 100 mg/dL to calculate it.

**Table 2:** Presenting serum glucose, sodium, tonicity, and corrected sodium levels in reported hyperglycemic crises.

	**DKA**	**HHS**	**CKDH**
Number of cases	7,812	755	772
**[Glu], mmol/L (mg/dl)**			
Mean^a^	31.4 (566)	57.4 (1,034)	43.4 (781)
Range^a^	18.4–143.3 (332–2,580)	17.2–146.4 (310–2,636)	25.8–188.9 (465–3,400)
Mean^b^	31.2 (562)	54.0 (972)	42.4 (763)
Range^b^^,^^c^	18.4–60.4 (332–1,087)	42.9–64.8 (773–1,166)	42.9–65.2 (497–1,174)
**Actual [Na], mmol/L**			
Mean^a^	133.7	145.8	128.1
Range^a^	113–164	116–191	92–147
Mean^b^	133.7	145.4	128.3
Range^b^^,^^c^	121–149	137–153	124–133
**Tonicity, mOsm/L**			
Mean^a^	299	349	300
Range^a^	256–394	322–422	257–463
Mean^b^	299	345	299
Range^b^^,^^c^	267–338	328–362	288–316
**Corrected [Na], mmol/L**			
Mean^a^	141.1	160.8	139.0
Range^a^	126–187	144–198	111–190
Mean^b^	141.0	160.8	138.5
Range^b^^,^^c^	127–159	151–167	135–143

*[Glu], serum glucose concentration; [Na], serum sodium concentration; DKA, diabetic ketoacidosis; HHS, hyperosmolar hyperglycemic state; CKDH, hyperglycemia in stage 5 chronic kidney disease. Means represent values weighed for the number of observations in each report*.

a *Means and ranges of all reports*.

b *Means and ranges of reports with ≥10 cases*.

c *Range of mean values*.

In Table 2, there exists considerable overlap of [Glu], [Na] tonicity, and corrected [Na] ranges in the three categories of hyperglycemia. DKA combined with HHS occurred in many instances. The term Diabetic Hyperosmolar Ketoacidosis (DHKA) was proposed for DKA combined with HHS (52). Patients on dialysis who presented with hyperglycemia and elevated corrected [Na] have usually lost hypotonic fluids through hemodialysis (252), or peritoneal dialysis (253–256, 274), with high glucose concentration in the dialysate.

The second important finding in Table 2 is in the mean corrected [Na] values. In DKA the overall mean corrected [Na] was within the normal range of [Na] (137–143 mmol/L). Among DKA series with ≥10 cases, mean corrected [Na] was in the eunatremic range in 45 series (66–69, 71, 74, 75, 80, 83, 84, 90, 91, 93, 104–106, 108, 109, 112, 113, 118, 120, 122, 125–127, 129, 135, 137, 138, 140, 143–146, 151, 155, 156, 158, 160, 163–165, 168–171) reporting 6,355 episodes, including the pivotal PECARN study in which mean corrected [Na] was 140.8 mmol/L in one study group and 140.7 mmol/L in each of the other three study groups (163); and in the hypernatremic range in the remaining 18 series (74, 76, 78, 80, 85, 91, 93, 98, 100–102, 104, 116, 120, 129, 133, 139, 169) reporting 1,301 episodes. Thus, although many patients have water deficits in excess of sodium and potassium deficits, an equal or even larger number of patients do not have excessive water deficits at presentation with DKA. This finding has important consequences in the choice of the tonicity of replacement solutions. In the HHS group, the mean corrected [Na], at > 160 mmol/L, is in the range of [Na] that constitutes a medical emergency (7). Mean corrected [Na] was in the eunatremic range in hyperglycemia of patients with CKD stage 5.

## Discussion

### Importance of the Corrected [Na] in Hyperglycemic Syndromes

Preventing cerebral edema is a key concern during treatment of hyperglycemic crises. Tonicity-related parameters have received attention in the studies of the pathogenesis of this complication. In 100 cases of cerebral edema developing during treatment of DKA, weighed means at presentation were as follows: [Glu] 34 mmol/L (612 mg/dL), [Na] 132.4 mmol/L, tonicity 299 mOsm/L, and corrected [Na] 140.6 mmol/L (72, 73, 94–96, 98, 102, 106, 109, 112, 117, 123, 124, 126, 131, 138). These values do not differ substantially from the mean values of all DKA cases in Table 2. However, factors related to tonicity statistically associated with brain edema during treatment of DKA include decrease in tonicity, large early infusion volumes, very high [Glu] at presentation, rapid decline in [Glu], very low [Na], and administration of large doses of insulin (102, 106, 118). The change in corrected [Na] during treatment of DKA was the best discriminator for the development of severe coma in one study (126). Deterioration of neurological manifestations associated with substantial rises of the corrected [Na] has been reported during treatment of both DKA (2, 126) and HHS (205, 223, 231).

Other reported factors associated with cerebral edema in DKA include the degree of acidosis (96, 106, 118, 278), high levels of blood urea at presentation (118, 277), and vasogenic factors (112). One study found no effect of the rate of replacement fluid infusion (138). The PECARN study found no significant differences in neurological manifestations during and following treatment of DKA between using 0.9% saline and 0.45% saline as replacement solutions and between fast and slow initial infusion rates (163). Vascular endothelial changes caused by elevated blood levels of cytokines and chemokines secondary to inflammatory status associated with DKA were proposed by the authors of the PECARN study as the main mechanism for the development of cerebral edema.

High value of corrected [Na] at presentation with DKA is associated with increased incidence and severity of acute kidney injury (AKI) (168, 279). Weighed mean values at presentation with DKA and AKI were 36.9 mmol/L (665 mg/dL) for [Glu], 135.5 mmol/L for actual [Na], 312 mOsm/L for tonicity, and 146.7 mmol/L for corrected [Na] in 93 patients (2, 92, 97, 121, 134, 142, 143, 158, 168). AKI occurs frequently in HHS (3, 213, 217, 218, 223, 227, 244).

### Management of Hyperglycemic Hypertonicity

#### Tonicity Targets During Treatment of Hyperglycemic Crises

Attention to tonicity plays a role in prevention of severe neurological manifestations during treatment of hyperglycemic emergencies. Decrease in tonicity from extracellular solute loss leads to osmotic entry of fluid into cells and could contribute to the development of cerebral edema (278). For this reason, one report proposed a very slow decrease in tonicity during the early stages of treatment (280). The optimal rate of decline in tonicity, however, has not been clarified. The proposed guideline for the maximal rate of decline in osmolality (tonicity should be targeted instead of total osmolality) during treatment of hyperglycemia is 3 mOsm/kg hourly (22, 23). One set of guidelines proposed a 3–8 mOsm/L range in hourly rate of decrease in tonicity (31).

The change in tonicity due exclusively to correction of hyperglycemia has two components, a fall in [Glu] and a rise in [Na]. Guidelines propose hourly rates of 2.8–4.2 mmol/L (50–75 mg/dL) for the decrease in [Glu] (1) and of 0.5 mmol/L for the commensurate rise in [Na] (30). Note that Katz's Δ[Na]/Δ[Glu] coefficient predicts a 0.8–1.2 mmol/L increase in [Na] for each 2.8–4.2 mmol/L decrease in [Glu].

#### Use of the Corrected [Na] During Treatment of Hyperglycemic Crises

The corrected [Na] predicts the relation between effective body solute and total body water after decrease of [Glu] to its desired level (2, 17) and should be used as a guide for the composition of replacement solutions in the same fashion as actual [Na] values are used to guide fluid management of dysnatremias (7, 281–285). Evidence presented earlier supports the use of the Al-Kudsi formula for calculation of the corrected [Na]. Two limitations of the corrected [Na] should be addressed during treatment: First, the corrected [Na] using the Al-Kudsi formula is not accurate in some conditions, mainly in advanced extracellular volume disturbances. Second, and more importantly, the corrected [Na] reflects the relation between effective body solute and body water at the moment of blood sampling (2, 17, 36). Correction of the extracellular volume deficit improves renal function and in the face of persistent hyperglycemia leads to large volume osmotic diuresis, which causes further water deficit and rises in the corrected [Na] (2).

We propose the following scheme for use of the corrected [Na] during treatment of hyperglycemic crises: The initial measurement of serum values should include osmolality in addition to basic metabolic panel. In the absence of an exogenous solute (e.g., ethanol) an osmol gap, that is the difference between measured osmolality and osmolarity calculated as 2 × [Na] + [Glu] + serum urea, where [Glu] and urea are in mmol/L, exceeding 10–12 mOsm/L indicates either presence of another non-dissociated compound in the serum (e.g., acetone) or a condition causing decrease in the water fraction of the serum (e.g., hyperlipidemia, hyperproteinemia). In the second case, falsely low [Na] values are reported when this measurement is performed in an autoanalyzer that requires dilution of the samples measured (286). If there is a large osmol gap, [Na] should be measured again in an apparatus that does not require dilution of the measured specimen, e.g., a blood gas machine, to obtain an accurate estimate of the presenting tonicity.

The tonicity of replacement solutions should be based on repeated calculations of the corrected [Na]. If the corrected [Na] at presentation is in the eunatremic range, infusion of isotonic saline should be started at a rate dictated by clinical manifestations of hypovolemia. Prevention of either decline or rise in the corrected [Na] is critical. Patients with corrected [Na] values within the normal range of [Na], like the average patient with DKA (Table 2), do not have relatively larger deficit of water compared to monovalent cations. In these patients, use of isotonic solutions as initial treatment of DKA and slow decline of [Glu], as proposed in the guidelines (1), leads to rapid correction of severe extracellular volume deficits and prevents sharp changes in the corrected [Na].

In subjects with initial corrected [Na] in the eunatremic range, tonicity should decline at a low rate. Maintenance of the corrected [Na] at the same level and decrease in [Glu] at the rate proposed in the guidelines (2.8–4.2 mmol/L hourly), will lead to a 0.8–1.2 mmol/L per hour rate of increase in [Na] and, from Equation (1), to a 1.2–1.8 mOsm/L per hour rate of decrease in tonicity. This conservative rate of decline in tonicity, which is slower than the hourly rate of 3 mOsm/L proposed in guidelines, may assist in preventing cerebral edema. In the rare instance of low presenting corrected [Na], or for treatment of cerebral edema, hypertonic saline infusion may be used (111). During treatment, urine volume should be monitored and [Glu], [Na], serum potassium concentration, and other relevant parameters should be measured frequently, initially every 1–2 h. The corrected [Na] should be calculated after each measurement of [Glu] and [Na] and should guide changes in the tonicity of the infusate. Development of large osmotic diuresis may lead to increases in the corrected [Na] and the need for hypotonic infusions later in the course of treatment.

A corrected [Na] in the hypernatremic range at presentation with hyperglycemia indicates excessive water deficit that must be corrected. Initially, infusion of isotonic fluids will correct rapidly volume deficits and will also decrease the level of hypertonicity. However, the subsequent development of large volume osmotic diuresis may lead to rise in the corrected [Na]. Monitoring urine volume, frequent measurement of the relevant serum biochemical values, and repeated calculation of the corrected [Na] after each measurement of [Glu] and [Na] is imperative. The corrected [Na] should not rise further; however, deciding whether it should remain at the same level at least early during the decrease in [Glu] or it should decrease at a slow rate (e.g., by 0.5 mmol/L every 1 or 2 h) from the start of the treatment requires prospective studies. Infusion of hypotonic solutions will eventually be needed regardless of whether the early phase of treatment aims at maintaining or decreasing the corrected [Na]. Addition of potassium salts to the infused saline should be guided by repeated measurements of the serum potassium concentration. In deciding the concentration of sodium in the replacement solutions, it is important to take into account the concentration of potassium salts in the infusate (2).

## Conclusions

The corrected [Na] calculated by the Al-Kudsi formula should guide the tonicity of replacement solutions. This use should be tempered by the knowledge that rarely encountered extreme volume disturbances can cause [Na] changes substantially different from those predicted by the corrected [Na] and, more importantly, that the corrected [Na] can vary greatly during treatment depending on changes in the external balances of water, sodium and potassium. For these reasons, frequent measurements of [Glu] and [Na], repeated calculation of the corrected [Na] after each measurement, and changes in the tonicity of replacement solutions based on the corrected [Na] are critical steps in the management of tonicity issues in hyperglycemia.

## Data Availability Statement

Publicly available datasets were analyzed in this study. This data came from tables of publications cited in the text.

## Author Contributions

TI: conceptualization. TI, KG, GB, CA, and AT: literature review. TI, GB, SL, and AT: methodology. SL, CA, and AT: visualization. TI and AT: writing-original draft preparation. KG, GB, SL, EA, and CA: writing-review and editing. All authors contributed to the article and approved the submitted version.

## Conflict of Interest

The authors declare that the research was conducted in the absence of any commercial or financial relationships that could be construed as a potential conflict of interest. The reviewer DM declared a past co-authorship with several of the authors TI, AT, and CA to the handling editor.
